# ‘It is Time to Prepare the Next patient’ Real-Time Prediction of Procedure Duration in Laparoscopic Cholecystectomies

**DOI:** 10.1007/s10916-016-0631-1

**Published:** 2016-10-14

**Authors:** Annetje C. P. Guédon, M. Paalvast, F. C. Meeuwsen, D. M. J. Tax, A. P. van Dijke, L. S. G. L. Wauben, M. van der Elst, J. Dankelman, J. J. van den Dobbelsteen

**Affiliations:** 1 Department of BioMechanical Engineering, Faculty of Mechanical, Maritime and Materials Engineering, Delft University of Technology, Mekelweg 2, 2628 CD Delft, The Netherlands; 2 Pattern Recognition Laboratory, Delft University of Technology, Mekelweg 4, 2628 CD Delft, The Netherlands; 3 Department of Surgery, Reinier de Graaf Groep, Reinier de Graafweg 3-11, 2625 AD Delft, The Netherlands

**Keywords:** Operating room scheduling, Procedure duration, Real-time, Prediction, Pattern recognition

## Abstract

Operating Room (OR) scheduling is crucial to allow efficient use of ORs. Currently, the predicted durations of surgical procedures are unreliable and the OR schedulers have to follow the progress of the procedures in order to update the daily planning accordingly. The OR schedulers often acquire the needed information through verbal communication with the OR staff, which causes undesired interruptions of the surgical process. The aim of this study was to develop a system that predicts in real-time the remaining procedure duration and to test this prediction system for reliability and usability in an OR. The prediction system was based on the activation pattern of one single piece of equipment, the electrosurgical device. The prediction system was tested during 21 laparoscopic cholecystectomies, in which the activation of the electrosurgical device was recorded and processed in real-time using pattern recognition methods. The remaining surgical procedure duration was estimated and the optimal timing to prepare the next patient for surgery was communicated to the OR staff. The mean absolute error was smaller for the prediction system (14 min) than for the OR staff (19 min). The OR staff doubted whether the prediction system could take all relevant factors into account but were positive about its potential to shorten waiting times for patients. The prediction system is a promising tool to automatically and objectively predict the remaining procedure duration, and thereby achieve optimal OR scheduling and streamline the patient flow from the nursing department to the OR.

## Introduction

Optimization of efficiency in healthcare is crucial to ensure a viable healthcare system for the future [1–4]. The Operating Room (OR), which is the most cost-intensive place of the hospital, is an area of particular interest with respect to efficiency measures [4–6]. Several studies have stated that many factors have to be taken into account in order to achieve optimal use of hospitals’ surgical capacities [4, 7–10]. Factors such as personnel and equipment resources, the time to prepare patients and unplanned emergency surgeries influence the daily planning of an OR complex. Moreover, the importance of reliable predictions of surgical procedure durations to achieve optimal OR scheduling has been emphasized [7–9, 11]. Surgical procedures that take longer than expected induce successive scheduled procedures to be postponed or cancelled. Furthermore, this causes undesired longer waiting times for patients, recurrent communication between the nursing staff and patients (and accompanying family), and an overload of the preoperative holding area [12]. On the contrary, procedures that finish earlier than expected can cause ORs to remain unused and OR teams to be unnecessarily waiting for the next patients [7]. Both scenarios are undesirable.

OR schedules are often based on estimates of surgical procedure durations, and do not account for variability in patient parameters or even the composition of the surgical team. Therefore, OR schedulers cannot fully rely on these estimated procedure durations [8] and need other methods to adapt their OR schedule as the day progresses. Typically, visual inspection is used to be informed about the progress of a procedure. However, the progress is not always easily recognizable and the scheduler needs to be familiar with many types of procedures. The alternative is to use verbal communication with the OR staff, through phone calls or physical presence in the OR, for asking an estimate of the remaining procedure duration. This causes disruptions of the surgical process and compromises safety in the OR [13, 14], which obviously should be avoided. The patient flow is currently regulated by an OR team member who calls the preoperative holding area to start the preparation of the next patient for surgery. Additionally, the estimated remaining procedure duration is based on the personal experience and routines of the OR staff. This lack of objective measurements adds complexity to the task of OR schedulers as they need to interpret the various situations and opinions in order to adapt and optimize the OR schedule. It also directly influences the patient’s experience in the hospital. A recent study has shown that the timing to start preparing the next patient was not optimal for laparoscopic cholecystectomies leading to a large variation in preparation and waiting times in the preoperative holding area (average of 47 min, with a standard deviation of 17 min) [15]. Besides, prompt changes in personnel or dealing with less experienced OR schedulers may have consequences for the efficient allocation of ORs as well as for the waiting times of patients. Gaining insight in the progress of surgical procedures and providing automatic and objective updates of the remaining procedure duration is of importance to achieve optimal OR scheduling and optimal patient flow.

The usage of devices and instruments can provide essential information about the progress of a procedure [11, 16–22]. Patterns in the usage of devices and instruments can be detected for various types of procedures. These patterns can then be used to detect the actual phase of a surgical procedure. Several pattern recognition approaches explored in previous studies have presented the potential of automatic recognition of the phase of procedures. However, there are many limitations regarding the application in real-time as the data of the entire procedure must be available [17, 19]. Other methods have shown to be usable for real-time applications but these required signals of numerous devices and instruments that currently cannot be obtained automatically [11, 19]. Automatic detection of these signals is however feasible by using techniques such as image analysis of laparoscopic videos [21, 23, 24], RFID technology [25, 26] or a multi-sensor surgical instruments table [27], but it is not yet implemented in daily practice. As far as the authors know, no system based on pattern recognition for prediction of end time of surgery has yet been implemented and tested in an OR.

In this study, we aim to monitor the progress of the procedure with one single piece of equipment, for simplicity and practical purposes. The challenge is to automatically obtain predictions of the end time rather than modelling all phases of a procedure and to provide an advice to the OR staff about the optimal timing to prepare the next patient. The goal of this study is: 1) to develop a real-time prediction system for the remaining procedure duration, based on methods that we presented in [15], and 2) to test the prediction system for reliability and usability in an OR.

## Material and methods

### Monitoring the activation of electrosurgical device

**Fig. 1:**
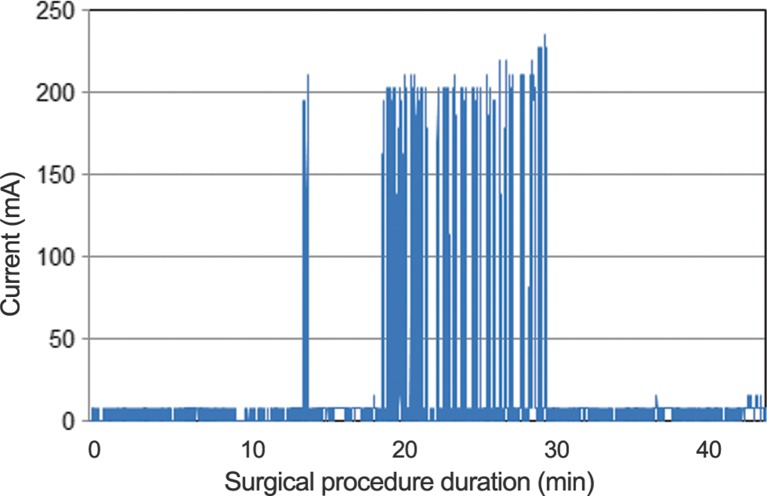
Example of the activation pattern of the electrosurgical device during an entire procedure

In laparoscopic cholecystectomy procedures, the electrosurgical device is activated during the removal of the gallbladder from the liver, which matches a certain stage of the procedure. Therefore, the activation of the electrosurgical device is suited to monitor for pattern recognition purposes. Activations of the electrosurgical device were detected with a current sensor. The amount of current delivered to the device was logged approximatively 10 times per second. Each peak in the amount of current corresponds to an activation of the device. This method is similar to the one presented in a previous conference paper [15]. An example of the activation pattern of the electrosurgical device during an entire surgical procedure is shown in Fig. 1.

### Pattern recognition

In [15], the activation pattern of the electrosurgical device was measured during 57 laparoscopic cholecystectomies performed by three different surgeons assisted by surgeons in training. The activation patterns of these procedures, with known end time, were used to train a classifier that classifies the data in two classes; procedures that are shorter and procedure that are longer than a certain amount of time. The Support Vector Machine (SVM) was selected as the best performing classifier and was chosen as algorithm for the prediction system. This classifier was trained using PRTools (statistical toolbox in MATLAB). More detailed information about the methods used can be found in our previous publication [15].

### Real-time prediction system for remaining procedure duration

**Fig. 2:**
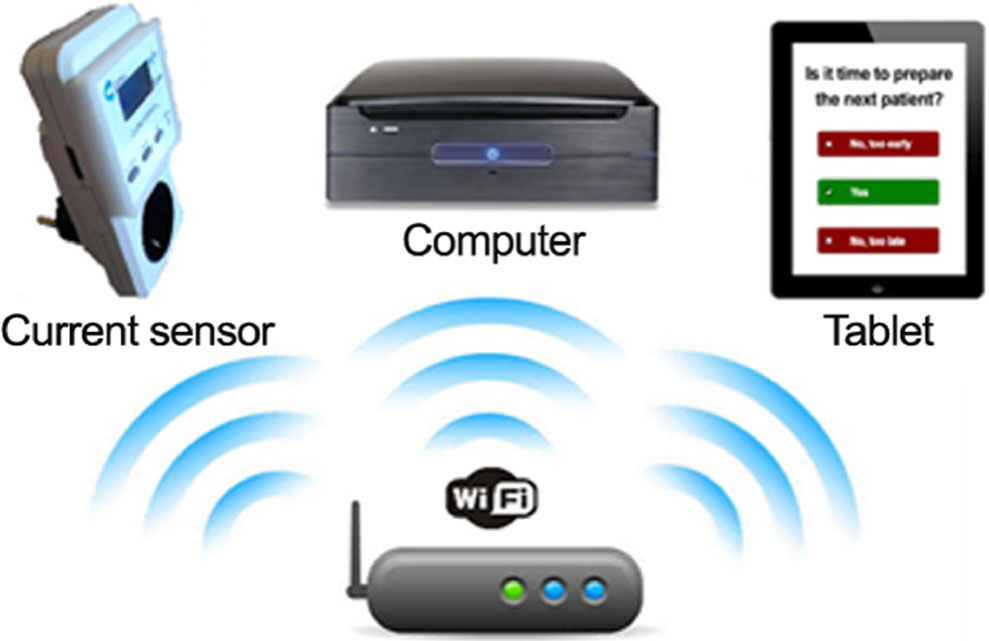
Schematic overview of the real-time prediction system

The classifier uses data of on-going surgical procedures in order to make a prediction automatically. Therefore, a real-time prediction system was developed (Fig. 2) that consisted of the following parts:

- *Server*. The central part of the system was a server storing all the data gathered from the different parts of the system in a Structured Query Language (SQL) database. The server also hosted a webpage that served as an interface to the users in the OR to start the system at the time of the first incision of the procedure. Afterwards, the webpage was also used to provide feedback about the timing to start preparing the next patient.
- *Current sensor*. The current sensor was connected with the server through Wi-Fi. As soon as the system was started, data were gathered and stored in a database.
- *Computer*. A computer extracted the data from the database, classified the procedure and obtained a prediction result. When it is time to prepare the next patient, the prediction is added to the database.
- *Tablet*. The webpage was updated when it was time to prepare the next patient. The webpage was accessed on a tablet, which was used to communicate the advice to the OR staff as well as to gather feedback about the usability of the prediction.

The optimal timing to start preparing the next patient was set 25 min before the last suture of the procedure. A margin of 10 min was considered as an acceptable prediction. These timings were based on observations in the preoperative holding area and discussions with OR staff. If after the first 15 min of measurements the prediction is such that the remaining procedure duration will be longer than 25 min, than the system will continue to measure data for another 5 min and a new prediction will be made. This process repeats until the system predicts it is time to start preparing the next patient or until 45 min have past.

### Accuracy of the system

The accuracy of the prediction system was tested during 21 laparoscopic cholecystectomies performed during 10 days. The procedures were performed by three different surgeons (the same three surgeons as for the training of the classifier) assisted by surgeons in training. The mean absolute error between the predicted and the actual total duration of a procedure was calculated. The accuracy of the prediction system was then compared with the mean absolute error of the OR staff’s predictions.

### Usability of the system

**Fig. 3:**
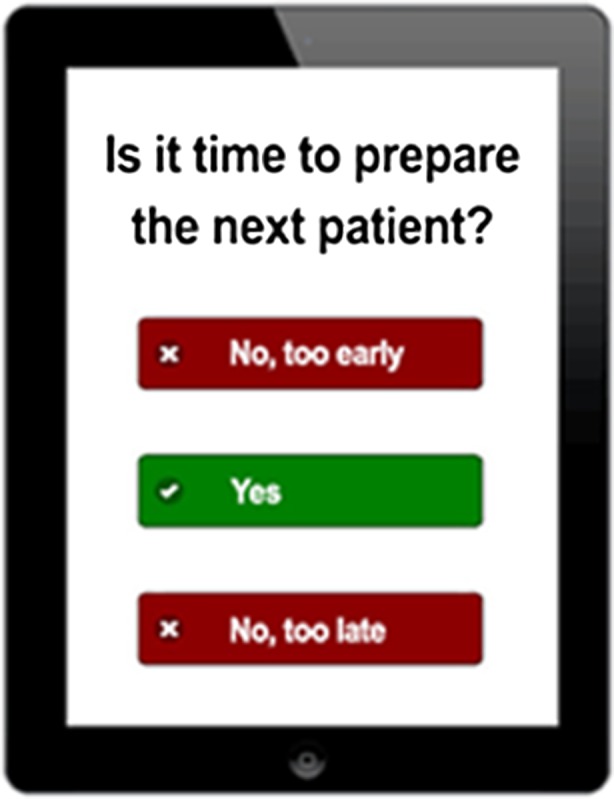
Webpage for gathering feedback of the OR staff

The usability of the prediction system was tested by gathering feedback from the OR staff when it was time to prepare the next patient according to the prediction system. This was done through the web-based interface asking if the timing was adequate, too early or too late (see Fig. 3). The answer was chosen according to the opinion of the surgeon and the nurse anaesthetist. After each day of testing, questions about the accuracy, benefits and potential utilisation were asked to the nurse anaesthetist, who is responsible for the phone call to start preparing the next patient.

## Results

### Accuracy of the system

**Fig. 4:**
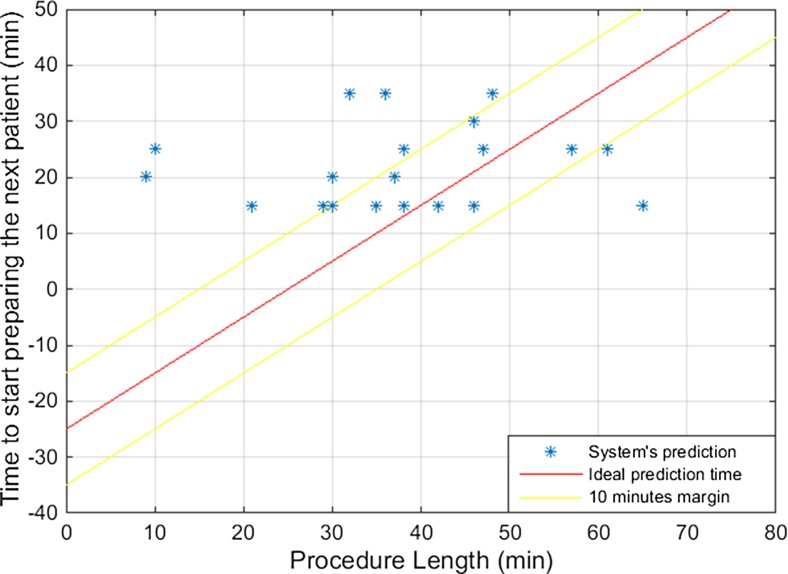
Timing to start preparing the next patient predicted by the prediction system

The accuracy of the prediction system is shown in Fig. 4. Ideal predictions would be placed along the red line representing the optimal timing to prepare the next patient, which was 25 min before the last suture. Predictions that were located above the red line were considered as being too late, while the ones located below the red line were considered as being too early. Predictions within the margin of 10 min of the ideal predictions, indicated in Fig. 4 between the two yellow lines, were considered as acceptable to be used in practice. The mean absolute error of the prediction system was 14 min.

**Fig. 5:**
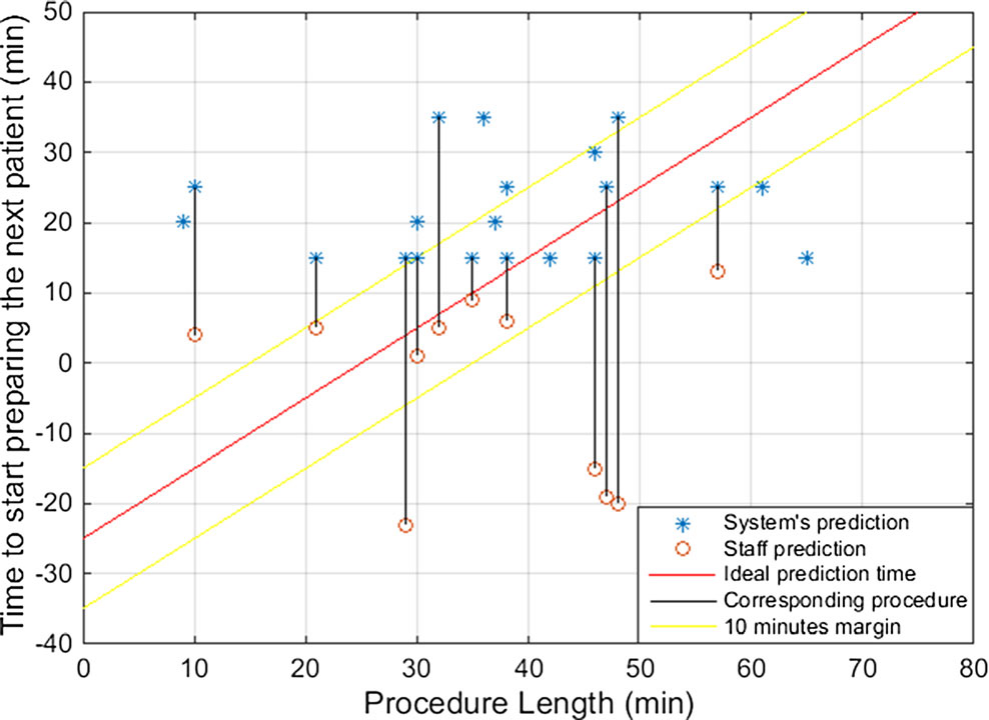
Timing to start preparing the next patient predicted by the prediction system and by the OR staff

The results of the prediction system and the corresponding predictions of the OR staff for each procedure are shown in Fig. 5. Predictions of the system and the OR staff corresponding to the same procedure are linked with a black line. However, not all system’s predictions presented in Fig. 5 have a corresponding OR staff’s prediction as some procedures were scheduled at the end of the day so no patient had to be prepared for a next surgery. The mean absolute error of the OR staff’s prediction was 19 min.

### Usability of the system

The feedback regarding the predicted timing to start preparing the next patient gathered through the web-based interface was as follows: one prediction was considered as too early, six were considered as being correct and fourteen as too late.

Nine out of the ten nurse anaesthetists completed the questionnaire at the end of each day of testing. The accuracy of the system was rated as ‘satisfactory’ by six respondents, as ‘unsatisfactory’ by two and one did not respond to this question. The benefits of the prediction system were rated as ‘satisfactory’ by three respondents and as ‘unsatisfactory’ by six. Furthermore, three respondents saw potential in the prediction system in the future, four did not and three respondents were undecided. The main doubts pointed out by the respondents regarded factors that could not be taken into account by technology, such as the performing surgeon, or the timing of lunchbreaks when the nursing area is understaffed and when the preparation of patients is taking longer. The main expected benefits of such a system are the shorter waiting times for patients and the support to inexperienced nurse anaesthetists.

## Discussion

This study presents a real-time prediction system for the remaining procedure duration that is based only on the activation of the electrosurgical device. This prediction system informs the OR staff about the optimal timing to start preparing the next patient. The reliability and usability of the system’s predictions were tested during 21 laparoscopic cholecystectomies. The mean absolute error was smaller for the prediction system (14 min) than for the OR staff (19 min). The system’s predictions were more reliable for procedures with average or long duration than for the ones with short duration. For the latter, there was not enough time for the prediction system to gather data and provide feedback to the OR staff as the initial predictions were made after the first 15 min of the procedure. For procedures longer than 40 min, the mean absolute error was 9 min and therefore within the margins of reliable predictions. For these procedures, the system’s predictions outperformed the OR staff’s predictions, which presented a mean absolute error of 29 min.

The timing to start preparing the next patient was mostly predicted later than optimal by the system and mostly earlier than optimal by the OR staff. As mentioned by Eijkemans et al., having ORs remaining unused for a while is undesirable [7] and the tendency of OR staff to start preparing the next patient earlier than needed is therefore understandable. It is preferable to have one patient waiting rather than an entire OR team. The prediction system focussed on obtaining minimal error but could be adjusted to lower the risk to have the OR team unnecessarily waiting.

The reliability reached in this study relied on the monitoring of only one piece of equipment and on a data of 57 laparoscopic cholecystectomies. Monitoring the use of additional equipment in the OR and gathering a larger dataset to train the prediction system is expected to further improve the reliability. Additional equipment in the OR could provide valuable information in order to improve the reliability of the predictions for short surgeries. Moreover, differences in surgical methods were detected between surgeons. Some clipped the cystic duct before activating the electrosurgical device, while others activated the device earlier. Training the prediction system for each individual surgical approach is expected to improve the reliability as well.

The opinions of the OR staff regarding the usability of the system varied. Some doubts were put forward about factors that could not be taken into account by such a prediction system, such as the surgeon’s speed and shortage of personnel at specific times of the day. However, the surgeon’s speed was recognized in the activation pattern of the electrosurgical device. Surgeons in training, who generally work slower than experienced surgeons, activated the electrosurgical device for a shorter time with longer intervals between activations. This type of pattern was recognized by the prediction system as a slower procedure. Dealing with shortage of personnel at specific times of the day was not incorporated in the prediction system but this type of information can easily be added to the prediction system in the future. According to the OR staff, the potential benefits of the prediction system were the shorter waiting times for patients and the support to inexperienced nurse anaesthetists. Nevertheless, the main benefit lays in the enhanced access to information on the progress of the procedure from outside the OR. This information can be used by the OR schedulers without having to interrupt the surgical process. Additionally, information on the progress of the procedure is valuable for the nursing staff, who can anticipate the preparation and transport of patients from and to the nursing department [12]. It can also reduce the efforts of the nursing staff to update the persons accompanying patients about their progress.

To conclude, the activation of the electrosurgical device was used to predict automatically and objectively the remaining duration in laparoscopic cholecystectomy procedures with a reasonable accuracy. Therefore, it is a promising prediction system to achieve optimal OR scheduling and optimal patient flow from the nursing department to the OR.
